# The Association Between Web-Based or Face-to-Face Lifestyle Interventions on the Perceived Benefits and Barriers to Exercise in Midlife Women: Three-Arm Equivalency Study

**DOI:** 10.2196/10963

**Published:** 2019-08-21

**Authors:** Amanda Mary McGuire, Charrlotte Seib, Janine Porter-Steele, Debra Jane Anderson

**Affiliations:** 1 Menzies Health Institute Queensland Griffith University Southport Australia; 2 Wesley Hospital Brisbane Australia

**Keywords:** exercise, physical activity, women, health behavior, behavioral medicine, health promotion, digital health, benefits and barriers

## Abstract

**Background:**

Noncommunicable diseases pose a significant threat to women’s health globally, with most diseases being attributed to modifiable risk factors such as physical inactivity. Women perceive a range of benefits and barriers to exercise; however, there is little evidence about the effect of different lifestyle intervention delivery modes on perceptions of exercise.

**Objective:**

This study aimed to compare the effect of a multiple health behavior change (MHBC) intervention called the Women’s Wellness Program. This intervention was delivered in 3 different modes on perceived exercise benefits, perceived exercise barriers, and actual physical activity and exercise in midlife women.

**Methods:**

Women aged 45 to 65 years were recruited via the study website. They were assigned in blocks to 3 different treatment groups (A: Web-based independent; B: face-to-face with nurse consultations; and C: Web-based with virtual nurse consultations). All participants received the 12-week intervention that utilizes principles from social-cognitive theory to provide a structured guide to promote healthy lifestyle behaviors with an emphasis on regular exercise and healthy eating. Data were collected using a self-report Web-based questionnaire at baseline (T1) and postintervention (T2) including perceived exercise benefits and barriers and exercise and physical activity. A data analysis examined both within- and between-group changes over time.

**Results:**

Participants in this study (N=225) had a mean age of 50.9 years (SD 5.9) and most were married or living with a partner (83.3%, 185/225). Attrition was 30.2% with 157 participants completing the final questionnaire. Women in all intervention groups reported a significant increase in positive perceptions of exercise (*P*<.05); a significant increase in exercise and overall physical activity (*P*<.01) with moderate-to-large effect sizes noted for overall physical activity (*d*=0.5 to *d*=0.87). Participants receiving support from registered nurses in the face-to-face and Web-based groups had a greater magnitude of change in benefit perceptions and physical activity than those in the Web-based independent group. There was no significant change in exercise barrier perceptions within or between groups over time.

**Conclusions:**

The results of this study suggest that the (MHBC) intervention is effective in increasing exercise benefit perceptions, overall physical activity, and exercise in midlife women. Although Web-based programs are cost-effective and flexible and can be delivered remotely, providing a range of options including face-to-face group delivery and personalized electronic health coaching from registered nurses has the potential to enhance participant engagement and motivation.

## Introduction

### Background

Noncommunicable diseases (NCDs) pose a significant threat to women’s health globally [1,2]. Recent estimates suggest that 4 NCDs including cardiovascular disease, cancer, respiratory disease, and type 2 diabetes account for the majority of premature deaths in women between the ages of 30 and 70 years [3]. Although it is clear that regular exercise has many physical and mental health benefits and is an important component of good health, many health promotion programs fail to adequately address the often-correlated nature of many modifiable risk factors [4]. For example, physical inactivity is often associated with other modifiable lifestyle risk factors such as an unhealthy diet, tobacco smoking, and overweight and obesity, with many adults having multiple risk factors for NCDs [4,5].

Among women, midlife is a time when the risk of developing an NCD increases [3], particularly among those who do not adhere to recommended physical activity and healthy eating guidelines [6,7] and often experience menopause-related weight gain [8,9]. Although there is no established definition of *midlife*, women between the ages of 40 and 65 years have normally finished childbearing and experience a physiological transition to perimenopause and menopause. Many women in this age bracket continue in paid employment with the average age that Australian women intend to retire increasing over the last decade to 64.4 years in 2017 [10]. According to Mishra et al, midlife (particularly perimenopause) is also a sensitive period when the cumulative effects of unhealthy lifestyle behaviors have a greater impact on disease risk [11]. Therefore, engaging in regular physical activity, eating a healthy diet, and maintaining a healthy body weight (body mass index [BMI]=18.5-25.0) [8] in midlife are essential to reduce risk and ensure optimal health and well-being as women age.

There is evidence that multiple health behavior change (MHBC) interventions tailored for women are effective in changing behavior [12,13]. Furthermore, given the multiple and complex role demands and stressors reported by women in this age group [14], flexible health promotion interventions have the potential to yield greater success. Over the past decade, *Web-based* or *internet* interventions also show promising results in promoting physical activity and healthy eating [15]. Moreover, though Web-based interventions targeting multiple health behaviors are fewer in number, there is evidence that they can provide an effective, flexible, cost-effective means of promoting healthy lifestyle behaviors [16-19].

Despite the potential efficacy of MHBC interventions, women perceive a range of benefits and barriers to changing exercise behavior [20-22]. Research suggests that perceived benefits of exercise include physical health and fitness, improved mental health and stress reduction, and reduced risk of illness [23-25]. Women’s perception of barriers to exercise are often complex and relate to a range of personal, social, and environmental factors such as lack of time, motivation, family support and care-giving responsibilities, climate and physical safety [23-25]. Arguably, these perceptions are very important to consider when designing health promotion interventions, with evidence that benefit and barrier perceptions are correlated with actual exercise behavior change [23,26]. In relation to behavior change theory, the concepts of *perceived benefits* and *perceived barriers* equate to *positive outcome expectations* and *impediments to change* that influence *self-efficacy* beliefs as described by Bandura in the social cognitive theory [27].

### Women’s Wellness Program Intervention

To date, there is a paucity of evidence about how different intervention delivery modes effect benefit and barrier perceptions. In this context, the Women’s Wellness Program (WWP) is a 12-week MHBC intervention designed for midlife women (Multimedia Appendix 1), targeting a range of modifiable risk factors including regular physical activity and exercise and healthy eating [12]. The original WWP that included a paper-based journal and 2 face-to-face nurse consultations was evaluated in a previous randomized controlled trial (N=90), finding that the intervention was effective in increasing physical activity, decreasing smoking, BMI, and weight [12]. On the basis of the social cognitive theory [27], the WWP includes detailed health education to promote health literacy and knowledge about the benefits of regular physical activity and exercise and incorporates strategies to overcome impediments/barriers to change, including realistic goal setting and health coaching to promote self-efficacy for exercise. The original Program was designed to be delivered face-to-face in a community practice setting by registered nurses trained to deliver the intervention. In this study, the WWP was revised and adapted to be delivered as a Web-based self-directed program hosted on a purpose-built website. The website also includes a separate Web portal for health consultations. Furthermore, a revised Program book was published both as an eBook and a hard copy. In this study, participants receiving health coaching received 4 nurse consultations at 0, 4, 8, and 12 weeks. Results in relation to the effect of the intervention on the primary outcome measure of climacteric symptoms are reported elsewhere [28].

### Objectives

This study addresses secondary outcome measures, where the effect of the WWP intervention delivered in 3 different modes/arms (Web-based independent; face-to-face with nurse consultations; and Web-based with virtual nurse consultations) on perceived exercise benefits, perceived exercise barriers, and actual physical activity and exercise in midlife women is investigated.

## Methods

### Participants and Procedure

Participants were Australian women aged between 40 and 65 years. Details of study participants, recruitment procedures, inclusion/exclusion criteria, and attrition are described elsewhere [28]. In short, following media publicity about the study, participants were recruited across Australia via the study website. Women with an existing diagnosis of an NCD or without computer/internet access were excluded.

### Measures

A self-report Web-based questionnaire was used to collect data from participants at baseline (T1) and postintervention (T2), including (1) sociodemographic information (T1 only), (2) perceived exercise benefits and barriers [25], (3) exercise and physical activity (Seattle Physical Activity questionnaire) [29], and (4) height (cm) and weight (kg). The BMI was calculated by dividing the weight in kilograms by the height in meters squared (kg/m^2^) [30].

This paper presents the pre- and postintervention perceived benefits and barriers measured using the Exercise Benefits and Barriers Scale (EBBS) [25]. The EBBS is a 46-item instrument using a forced response Likert-type scale. The scale contains 29 benefit items summed to calculate a total benefit subscale score (EBBS_BEN_), with higher scores indicating higher benefit perceptions. Benefit items are then grouped and summed to create benefit subcategories: life enhancement, physical performance, psychological outlook, social interaction, and preventive health. Example benefit items include the following: 32. Exercise improves my self-concept; 15. Exercise increases my level of physical fitness; 2. Exercise decreases feelings of stress and tension; 11. Exercise lets me have contact with friends and persons I enjoy; and 13. Exercise will keep me from having high blood pressure.

The 46-item scale contains 14 barrier items summed to get a total barrier subscale score (EBBS_BAR_), with higher scores indicating higher barrier perceptions. Barrier items are grouped and summed to create barrier subcategories: exercise milieu, time expenditure, physical exertion, and family encouragement. Example barrier items include: 9. Places for me to exercise are too far away; 4. Exercising takes too much of my time; 37. Exercise takes too much time from my family responsibilities; 6. Exercise tires me; and 33. My family members do not encourage me to exercise.

The EBBS demonstrates good reliability and internal consistency in studies that investigate exercise benefits and barriers in women [20-22]. In this study, subscale reliability was calculated with a Cronbach alpha of .94 for the benefit subscale and .87 for the barrier subscale, indicating high internal consistency.

Additional anecdotal feedback about what participants liked and disliked about the Program was obtained and invitation to make *other comments* was given through 3 open-ended questions asked postintervention via the Web-based questionnaire.

### Intervention

The participating women completed baseline questionnaires before being assigned in blocks to one of the 3 different treatment modality groups. The 12-week program utilizes principles from the social-cognitive theory [27] to provide participants with a structured guide to promote healthy lifestyle behaviors with an emphasis on regular physical activity and exercise, healthy eating, healthy weight, stress management, and health screening behaviors. In relation to exercise and physical activity, the intervention provides detailed evidence-based information in plain language about the current guidelines for physical activity [6]. Information about the multiple health benefits of regular physical activity (aerobic exercise, strength training, and stretching) on physical and mental health and the reducing risk of chronic disease is provided in the Program book and website and reinforced in nurse consultations. Photographic illustrations of strength exercises are provided with practical advice on starting and maintaining a regular physical activity schedule, with daily walking recommended as the starting activity for participants who are sedentary or unfit. Over the 12 weeks, participants are encouraged to gradually increase the frequency and intensity of physical activity and exercise, with Program 1 strength training exercise introduced in Week 2 and Program 2 strength exercises with dumbbells introduced in Week 5. The Program book and website include weekly activity planning with participants invited to identify and reflect on their barriers to exercise behavior change through journal activities, reflections, and discussions with a registered nurse. Participants receiving nurse consultations are supported to develop personalized goals for exercise and diet that are specific, measurable, achievable, relevant, and time bound at 0, 6, and 12 weeks.

The WWP intervention including the Program book, the Program website, and health consultations is delivered in 3 different formats: (1) *Web-based independent* (Arm A) had access to the WWP website that contains all of the information provided in the book and an electronic copy of the Program book only; (2) *Face-to-face group supported* (Arm B) included a hard copy of the Program book and 4 30- to 60-min face-to-face consultations provided by a registered nurse at 0, 4, 8, and 12 weeks; and (3) *Web-based supported* (Arm C) were able to access the WWP website, download an electronic copy of the Program book, and were also provided 4 virtual consultations through a portal built into the website at 0, 4, 8, and 12 weeks. Intervention fidelity was maintained through provision of structured facilitator training, through consistent record keeping and auditing, and by employing one registered nurse to deliver all consultations.

### Statistical Analysis

Analyses were performed using IBM® SPPS Statistics version 22 [31]. Descriptive data are expressed as counts and percentages or mean (SD), whereas inferential statistics were performed using *t* tests and analysis of covariance (ANCOVA) or their nonparametric equivalent. The statistical significance was set at alpha=.05. The effect size was also calculated using the Cohen *d* standard formula [32] to examine the meaning and magnitude of change in the 3 groups over the study period. Using Cohen guidelines [32], an effect size of 0.20 was deemed to be small, an effect size of 0.50 was moderate, and an effect size of 0.80 or more was considered to be large.

### Ethical Approval

Before recruitment and data collection, ethical approval was obtained from the relevant Human Research and Ethics Committee (QUT HREC Approval No: 1300000048). Participation was voluntary, and women were able to withdraw from the study at any time. Furthermore, participants did not receive any rewards or incentives for participation; however, they were able to retain the Program book and materials and have continued access to the website resources.

## Results

### Sociodemographic Characteristics and Baseline Body Mass Index and Physical Activity

Participants in this study had a mean age of 50.9 years (SD 5.9) and most were married or living in a de-facto relationship (83.3%, 185/225). Overall, 79.1% of women were Australian born, most worked either full- or part-time (53.0%, 119/225 or 29.5%, 66/225 respectively), and almost two-thirds (68.9%, 155/225) of participants were university educated. According to the World Health Organization categories [30], where overweight is classified as BMI 25.0 to 29.9 and obesity BMI greater than 30.0, many of the participating women reported being overweight (35.5%, 79/225) or obese (32.9%, 74/225). When asked about general daily activity levels (including housework, gardening, shopping, caring for children, or activity at work), the proportion of all participants at baseline who were very active was 1.8% (4/225); moderately active, 32.2% (72/225); mildly active, 46.2% (104/225); and sedentary, 19.1% (43/225). Reported levels of aerobic exercise (15 min at a time in the past month including brisk walking, jogging, swimming, and cycling) for all women at baseline found 21.8% (49/225) getting no aerobic exercise; 36% (81/225) one or 2 times/week; 27.1% (61/225) 3 or 4 times/week; 10.2% (23/225) 5 or 6 times/week; and 4.4% (10/225) daily exercise.

Although 225 women completed the baseline questionnaire, an attrition rate of 30.2% (68/225) meant that 157 women completed the final questionnaire following the 12-week intervention. A comparison of retained participants and those lost to follow-up showed significantly more participants being lost from the Web-based independent Arm A (35.5%, 49/138) compared with Arm B (9.7%, 4/41) and Arm C (30.2%, 15/46). The comparison of baseline sociodemographic and health characteristics of the 3 intervention groups showed no significant within- or between-group differences [30].

**Figure 1:**
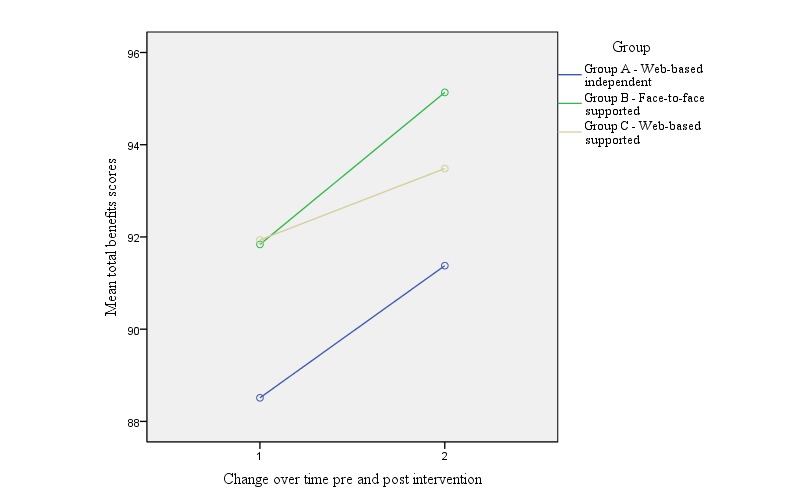
Change over time in average perceived benefits of exercise scores within and between groups.

**Figure 2:**
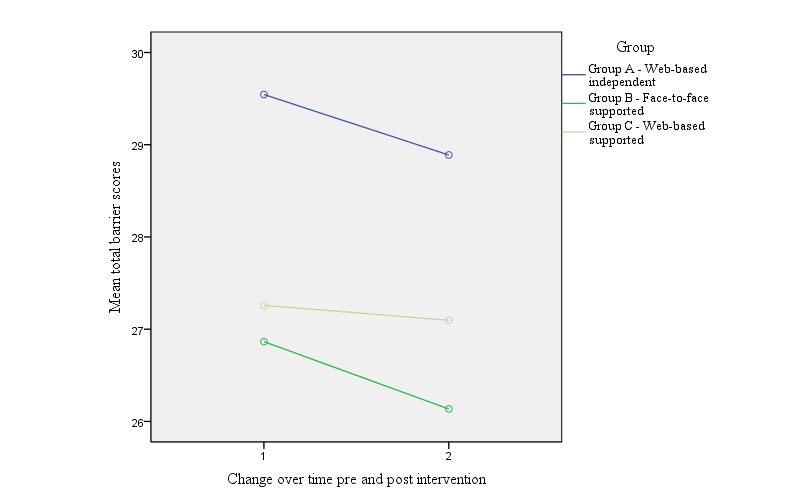
Change over time in average perceived barriers to exercise scores within and between groups.

Table 1 presents the mean total exercise benefits, barriers and benefits, and barriers subscale scores pre- and postintervention within each group. Although there was no statistically significant change in barrier scores over time, in contrast there was a significant increase in average total benefits, psychological, and social subscale scores within all 3 intervention groups. A one-way between-group ANCOVA showed no significant difference between the 3 intervention groups over time for both total exercise benefit scores (*F*_2,153_=0.30; *P*=.74; partial eta squared=0.004) and total exercise barrier scores (*F*_2,153_=0.65; *P*=.52; partial eta squared=0.01). Figures 1 and 2 present the change over time in average total perceived benefit and barrier scores within and between groups.

**Table 1:** Mean perceived benefits and barriers to exercise scores within and between groups pre- and postintervention (N=157). Mean scores are based on an ordinal scale representing the extent to which women strongly disagree/disagree/agree/strongly agree that the item is a benefit/barrier to exercise (range 1 to 4 with high scores representing higher agreement).

Variable		Arm A: Web-based independent (n=89), mean (SD)		Arm B: Face-to-face supported (n=37), mean (SD)		Arm C: Web-based supported (n=31), mean (SD)	
		Pre	Post	Pre	Post	Pre	Post
**Perceived benefits^a^**							
	Total benefits	88.5 (10.8)	91.4 (13.4)^b^	91.8 (9.5)	95.1 (14.4)^b^	91.9 (11.1)	93.5 (10.4)^b^
	Life enhancement	3.1 (0.5)	3.2 (0.5)	3.3 (0.4)	3.3 (0.5)	3.3 (0.5)	3.2 (0.4)
	Physical	3.4 (0.4)	3.4 (0.4)	3.4 (0.4)	3.4 (0.5)	3.5 (0.4)	3.4 (0.4)
	Psychological	2.9 (0.4)	3.2 (0.5)^b^	3.0 (0.3)	3.3 (0.6)^b^	3.0 (0.3)	3.4 (0.4)^b^
	Social	2.3 (0.6)	2.4 (0.7)^b^	2.5 (0.6)	2.7 (0.7)^b^	2.5 (0.6)	2.5 (0.5)^b^
	Preventive health	3.3 (0.5)	3.3 (0.5)	3.4 (0.4)	3.4 (0.5)	3.3 (0.6)	3.3 (0.4)
**Perceived barriers^c^**							
	Total barriers	29.6 (6.1)	29.0 (6.9)	26.9 (6.5)	26.1 (5.3)	27.3 (5.6)	27.1 (6.2)
	Exercise milieu	1.9 (0.5)	1.8 (0.5)	1.7 (0.5)	1.7 (0.4)	1.7 (0.5)	1.7 (0.5)
	Time expenditure	2.2 (0.6)	2.2 (0.6)	2.0 (0.6)	1.9 (0.5)	2.1 (0.5)	2.1 (0.7)
	Physical exertion	2.6 (0.6)	2.6 (0.6)	2.4 (0.6)	2.3 (0.5)	2.3 (0.5)	2.3 (0.5)
	Family encourage	1.8 (0.7)	1.9 (0.9)	1.7 (0.8)	1.7 (0.7)	1.7 (0.5)	1.7 (0.5)

^a^Exercise Benefits Subscale.

^b^Within-group paired *t* test; *P*<.05.

^c^Exercise Barriers Subscale.

Changes in overall physical activity, aerobic exercise, and general daily activity are presented in Table 2. There was a significant difference in all physical activity variables within all groups postintervention (*P*<.01). Between-group comparison of overall physical activity was close to statistical significance (*F*_2,148_=45.1; *P*=.052; partial eta squared=0.04), with a greater increase in face-to-face supported Arm B and Web-based supported Arm C compared with Web-based independent Arm A.

**Table 2:** Comparison of overall physical activity, aerobic exercise, and general daily activity within groups pre- and postintervention (N=157).

Variable		Arm A: Web-based independent (n=89)		Arm B: Face-to-face supported (n=37)		Arm C: Web-based supported (n=31)	
		Pre	Post	Pre	Post	Pre	Post
**Overall physical activity (PA)^a^**							
	Mean (SD)^b^	5.4 (1.8)	6.3 (2.1)^c^	5.8 (1.9)	6.9 (1.9)^c^	5.6 (1.9)	7.3 (1.5)^c^
	Median^b^	6.0	7.0^c^	6.0	8.0^c^	6.0	8.0^c^
**Weekly aerobic exercise** **, n** **(%)**							
	2 or less times weekly	55.7 (49)	33.7 (30)	59.5 (22)	24.3 (9)	64.5 (20)	29.0 (9)
	3-4 times weekly	31.8 (28)	36.0 (32)	21.6 (8)	35.1 (13)	25.8 (8)	29.0 (9)
	5+ times weekly	12.5 (12)	30.3 (27)	18.9 (7)	40.5 (15)	9.7 (3)	41.9 (13)
	McNemar test	—^d^	21.3^c^	—	14.3^c^	—	14.5^c^
**General daily activity** **,** **n** **(%)**							
	Sedentary	18.0 (16)	6.7 (6)	13.9 (5)	8.1 (3)	22.6 (7)	6.5 (2)
	Mildly active	49.4 (44)	36.0 (32)	52.8 (19)	29.7 (11)	35.5 (11)	22.6 (7)
	Moderately/very active	32.6 (29)	57.3 (51)	33.3 (12)	62.2 (23)	41.9 (13)	71.0 (22)
	McNemar test	—	25.7^c^	—	9.3^c^	—	8.6^c^

^a^Overall physical activity including exercise and general daily activity measured on a scale of 0 to 10.

^b^Paired *t* test and Wilcoxon Signed Rank test.

^c^*P*<.01.

^d^Not applicable.

### Effect Size

Table 3 presents results of the effect size analysis within and between groups over time in perceived benefits and barriers to exercise and overall physical activity. A small effect for perceived barriers to exercise was observed, with a small-to-moderate effect for perceived benefits to exercise within all 3 groups postintervention (Cohen *d*_*change*_). A moderate-to-large effect was seen in overall physical activity, within all intervention groups. Using Cohen *d*_*2*_ to compare the difference in effect size between Arm A (Web-based independent) and Arm B (face-to-face supported) and Arm C (Web-based supported), there was a small-to-moderate effect size observed for all variables.

To further illustrate and compare the magnitude of change over time within and between each of the 3 groups, Table 4 presents exercise benefits and barriers subscale variables and overall physical activity, grouped by effect size and study arm. Of note is the large effect size for psychological benefits in all groups and overall physical activity in the Web-based supported group C. There was a moderate effect size observed for overall physical activity in the Web-based independent (Arm A) and face-to-face supported (Arm B) groups.

**Table 3:** Effect size within and between groups over time in perceived benefits and barriers to exercise and overall physical activity (N=157).

Variables		Cohen *d*_change_^a^			Cohen *d*_2_^b^	
		Arm A: Web-based independent	Arm B: Face-to-face supported	Arm C: Web-based supported	A-B post	A-C post
**Perceived benefits^c^**						
	Total benefits	0.27	0.30	0.14	0.28	0.16
	Life enhancement	0.02	0.00	0.20	0.20	0.00
	Physical	0.00	0.00	0.25	0.00	0.00
	Psychological	0.70	1.0	1.3	0.20	0.40
	Social	0.21	0.33	0.13	0.43	0.14
	Preventive health	0.00	0.00	0.00	0.20	0.00
**Perceived barriers^d^**						
	Total barriers	0.10	0.11	0.03	0.42	0.28
	Exercise milieu	0.20	0.00	0.00	0.01	0.20
	Time expenditure	0.00	0.20	0.00	0.50	0.20
	Physical exertion	0.00	0.20	0.00	0.50	0.42
	Family encourage	−0.14	0.00	0.00	0.22	0.22
	Overall physical activity	0.50	0.60	0.87	0.31	0.48

^a^Cohen *d*_change_ compared the difference in the effect size within groups over time.

^b^Cohen *d*_2_ compared the difference in effect size between groups postintervention.

^c^Exercise Benefits Subscale.

^d^Exercise Barriers Subscale.

**Table 4:** Comparison of magnitude of change postintervention within each group for exercise benefits and barriers subscale scores and overall physical activity.

Effect size^a^	Arm A: Web-based independent	Arm B: Face-to-face supported	Arm C: Web-based supported
Large >.7	Psychological benefits	Psychological benefits	Psychological benefits; Overall PA
Moderate .4 to .7	Overall PA	Overall PA	—^b^
Small .2 to .4	Total benefits score; Social benefits; Exercise milieu barriers	Social benefits; Total benefits score; Time barriers; Physical exertion barriers	Physical benefits; Life enhancement benefits
Very small <.2	Family barriers; Total barriers score; Life enhancement benefits	Total barriers score	Total benefits score; Social benefits; Total barriers score

^a^Cohen d_change_ compared the difference in effect size with groups over time.

^b^Not applicable.

## Discussion

### Principal Findings

This study has reported the results of a 3-arm MHBC intervention on perceived exercise benefits and barriers and self-reported physical activity and exercise in midlife women. Postintervention, women in all 3 arms reported a significant increase in overall exercise benefit perceptions and increased physical activity.

With regard to exercise benefits, there was a significant change in perceptions about the psychological and social benefits in particular, with large effect sizes being noted. This change was associated with a significant increase in overall physical activity and exercise, with moderate-to-large effect sizes across all 3 groups. What is striking about these results is the proportion of participants who moved from lower levels of exercise to reporting regular exercise on 5 or more days per week postintervention. These changes in benefit perceptions and actual physical activity are likely to be a result of specific program content and strategies, including detailed health promotion information contained in the Program book, individualized goal setting, and weekly exercise planning activities undertaken by all participants over the 12 weeks of the trial.

When comparing the magnitude of change between groups over time, the Web-based independent group (Arm A) was used as the comparison group. In comparison with the Web-based independent group, both the face-to-face supported group (Arm B) and Web-based supported group (Arm C) had greater change in benefit and barrier perceptions and overall physical activity postintervention. Women who received face-to-face support reported moderately higher social benefits, with the Web-based supported group reporting moderately higher psychological benefits. This is likely to be attributed to the additional support that Arm B and C received through nurse consultation and health coaching and the peer support available in Arm B. These results suggest that personalized and tailored health consultation provided by registered nurses with knowledge and skills in health behavior change theory and communication can facilitate positive behavior change.

In support of this, anecdotal feedback from participants indicated that having the opportunity to discuss personal health issues and work and family commitments and discuss strategies for change with a supportive health professional was highly valued by women in the face-to-face and Web-based supported groups. In contrast, feedback from participants in the Web-based independent group highlighted the lack of support being a barrier to engagement with the program, and it is likely that this contributed to the higher attrition rate in this group. However, participants in the Web-based independent group who remained in the study reported significant increases in physical activity, indicating that for some women undertaking a Web-based intervention independently is an effective option for undertaking a behavior change intervention.

Interestingly, despite a reported increase in physical activity and positive benefit perceptions, there was no statistically significant change in the average exercise barrier perceptions within or between groups postintervention. A possible explanation is that the average exercise barrier perceptions in all groups at baseline were relatively low, perhaps reflecting the fact that participants were motivated volunteers who self-selected to enroll in a health promotion program.

### Comparison With Previous Work

In relation to the effect of a behavior change intervention on exercise benefit and barrier perceptions, there are limited studies to allow direct comparison. Our results are somewhat similar to a study on Latino American women that found an increase in total benefit perceptions following a 9-month biweekly education and exercise intervention [33]. In contrast, Kennedy et al [33] report a decrease in barrier perceptions. Other studies in women report no change in total benefit or barrier perceptions post exercise intervention [34,35]. One of these studies was a 7-week structured walking program designed for postmenopausal African American women [35] and the other, a 12-week group exercise program for mother and daughter pairs [34]. A more recent study investigated perceived benefits and barriers to exercise participation in *n*=43 overweight women with polycystic ovarian syndrome participating in a 3-arm 20-week lifestyle program, finding a significant improvement in increased benefit and decreased barrier perceptions [36]. Our study has appeared to be the first to report the effect of a 12-week MHBC intervention on exercise benefit and barrier perceptions in healthy midlife women.

There is a large body of literature in relation to the effect of Web-based physical activity interventions with our results aligning with systematic review [15] and meta-analysis [37] findings that indicate that the majority of internet PA interventions in adults lead to significant increases in physical activity. These studies both report that average effect sizes are usually small (*d*=0.14); in contrast, our study found moderate-to-large effect sizes across all intervention groups (*d*=0.5 to *d*=0.87, respectively). A possible explanation for these results is that the WWP intervention is specifically designed for midlife women with variable fitness levels and allows personal choice in type, intensity, and frequency of physical activity and exercise, facilitating incremental change over time. The intervention materials also explicitly address the multiple benefits of regular exercise for midlife women’s health and healthy ageing and provide practical strategies for overcoming barriers to change, perhaps enhancing participant motivation to exercise. This is consistent with evidence that client-centered and personalized lifestyle interventions with ongoing support are likely to be more effective in changing behavior in both the short and medium term [38]. Results in this study are also consistent with our previous findings [12], where the original WWP was tailored for midlife women and included personalized support.

Similar to studies comparing different intervention delivery modes [39,40], our study found increases in physical activity in all groups. A similar study by Steele et al [39] investigated the effectiveness of delivery modes for a 12-week pedometer-based behavior change program (Health-*e* Steps), comparing face-to-face, internet-mediated, and internet-only delivery with an equivalent magnitude of increase in physical activity over time being reported. In contrast, our study found a greater magnitude of change in PA in the face-to-face and Web-based supported groups compared with the Web-based independent group. In contrast to the Web-based independent group, the face-to-face and Web-based supported groups both received health coaching including goal setting from a registered nurse. Recent systematic reviews indicate that behavioral counselling to promote PA and a healthy diet [41] and setting goals [42] are effective and important in facilitating behavior change. Building on our previous work [12], the results in this study including qualitative feedback from participants suggest that *one size does not fit all*. In addition to the tailored and personalized nurse-led intervention, the ability to offer women the choice of intervention delivery modes to fit their motivation levels, family and work commitments, and preferences is a positive outcome of this project.

### Limitations

The results of this study need to be considered in light of the limitations. First, although women were recruited across Australia, volunteers were generally well-educated and from high-income groups. Although this is often the case for studies involving Web-based interventions with volunteers predominantly white, middle aged, and female [18], this is likely to limit the generalizability of results. In terms of attrition, average barrier scores were higher in the Web-based independent group (Arm A) that had the highest level of attrition (35.5%), so data collected postintervention did not include those participants. Furthermore, this study had no control group, largely because it was designed to investigate the equivalency of the different intervention arms. Without a control group, it is difficult to definitively attribute posttest changes to the effects of the intervention. The study relied on self-report data that can be prone to response bias, with participants more likely to report positive outcomes for behavior change measures such as physical activity. Furthermore, data collection took place immediately after the intervention, so long-term effects might be lower.

### Conclusions

Despite limitations, the results of this study suggest that the MHBC intervention (WWP) including personalized and tailored health coaching from registered nurses is likely to be effective in increasing exercise benefit perceptions, overall physical activity, and exercise in midlife women. The moderate-to-large effect sizes found in this study are particularly encouraging, indicating that for motivated participants, undertaking the program in different modes was beneficial in changing exercise behavior and positive perceptions of increased physical activity. Although Web-based interventions are cost-effective and flexible and can be delivered remotely, providing a range of options including face-to-face group delivery electronic health coaching from registered nurses has the potential to enhance participant engagement and motivation. With an increased focus on prevention of NCDs being evident, this study makes a timely contribution to the knowledge about exercise behavior change for prevention of NCDs in midlife women.

## Appendix

Multimedia Appendix 1 Women's Wellness Program Intervention.

## Abbreviations

ANCOVA: analysis of covariance
BMI: body mass index
EBBS: Exercise Benefits and Barriers Scale
MHBC: multiple health behavior change
NCD: noncommunicable disease
WWP: Women’s Wellness Program
