# Supplementary material for: The Association Between Web-Based or Face-to-Face Lifestyle Interventions on the Perceived Benefits and Barriers to Exercise in Midlife Women: Three-Arm Equivalency Study
Source: J Med Internet Res. 2019 Aug 21;21(8):e10963. doi: 10.2196/10963 (PMC6724500; doi:10.2196/10963)
Supplement: Multimedia Appendix 1 [file jmir_v21i8e10963_app1.pdf]

Effect of a web-based versus face to face lifestyle intervention of perceived benefits and barriers to exercise in midlife women: A three arm equivalency study.

Dr Amanda McGuire, Dr Charrlotte Seib, Dr Janine Porter-Steele, Professor Debra Anderson

THE WOMEN'S WELLNESS RESEARCH PROGRAM

MENZIES HEALTH INSTITUTE QUEENSLAND

Griffith UNIVERSITY

The Women's Wellness Program©

Healthy eating

Increase physical activity and exercise

Chronic disease risk factor reduction

Knowledge about menopause and health for women over 40

Optimise health and well-being

Intervention components

- Program website
- Program book – hard copy and e-book
- Health coaching delivered by Registered Nurses

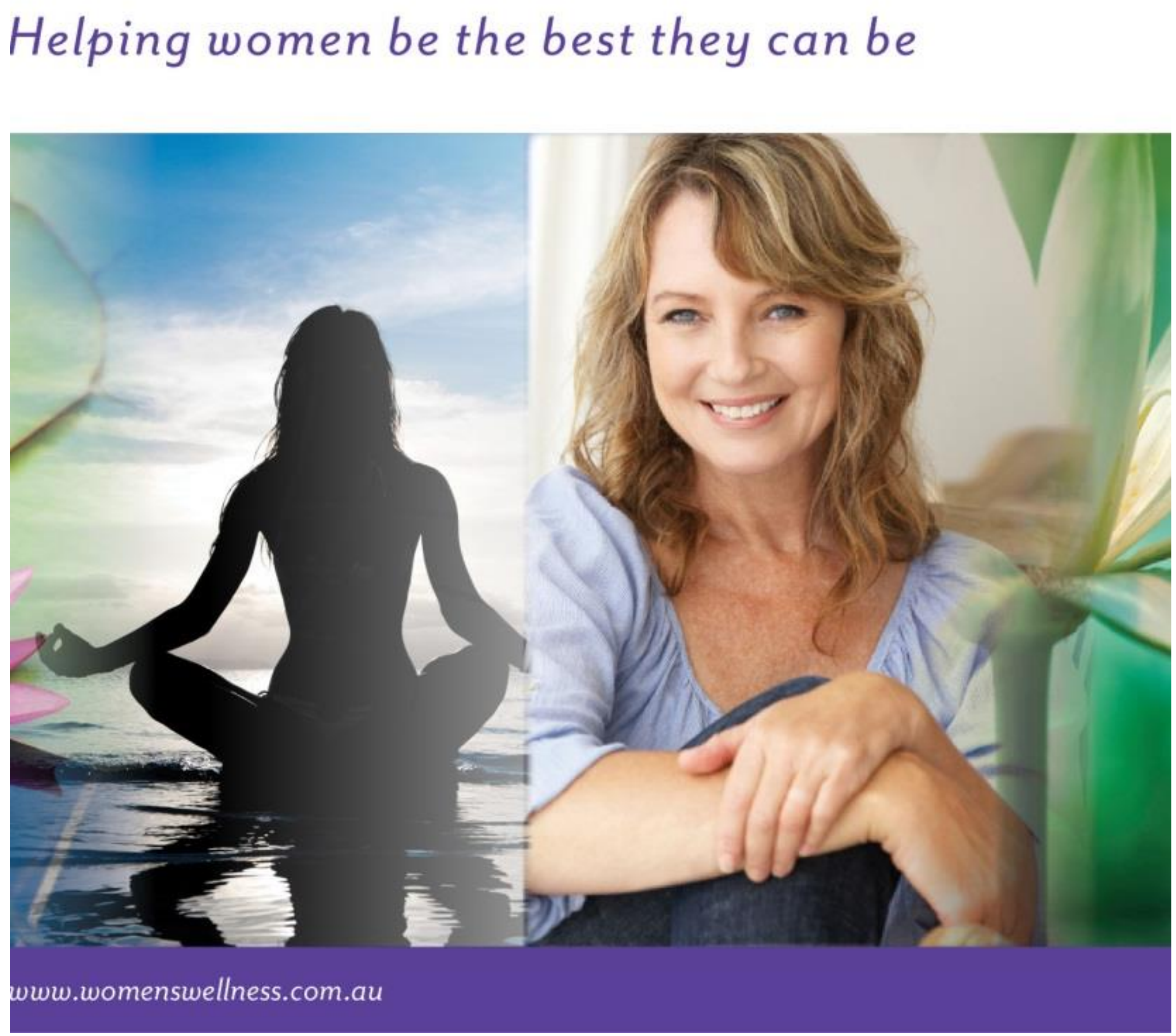

Website home page

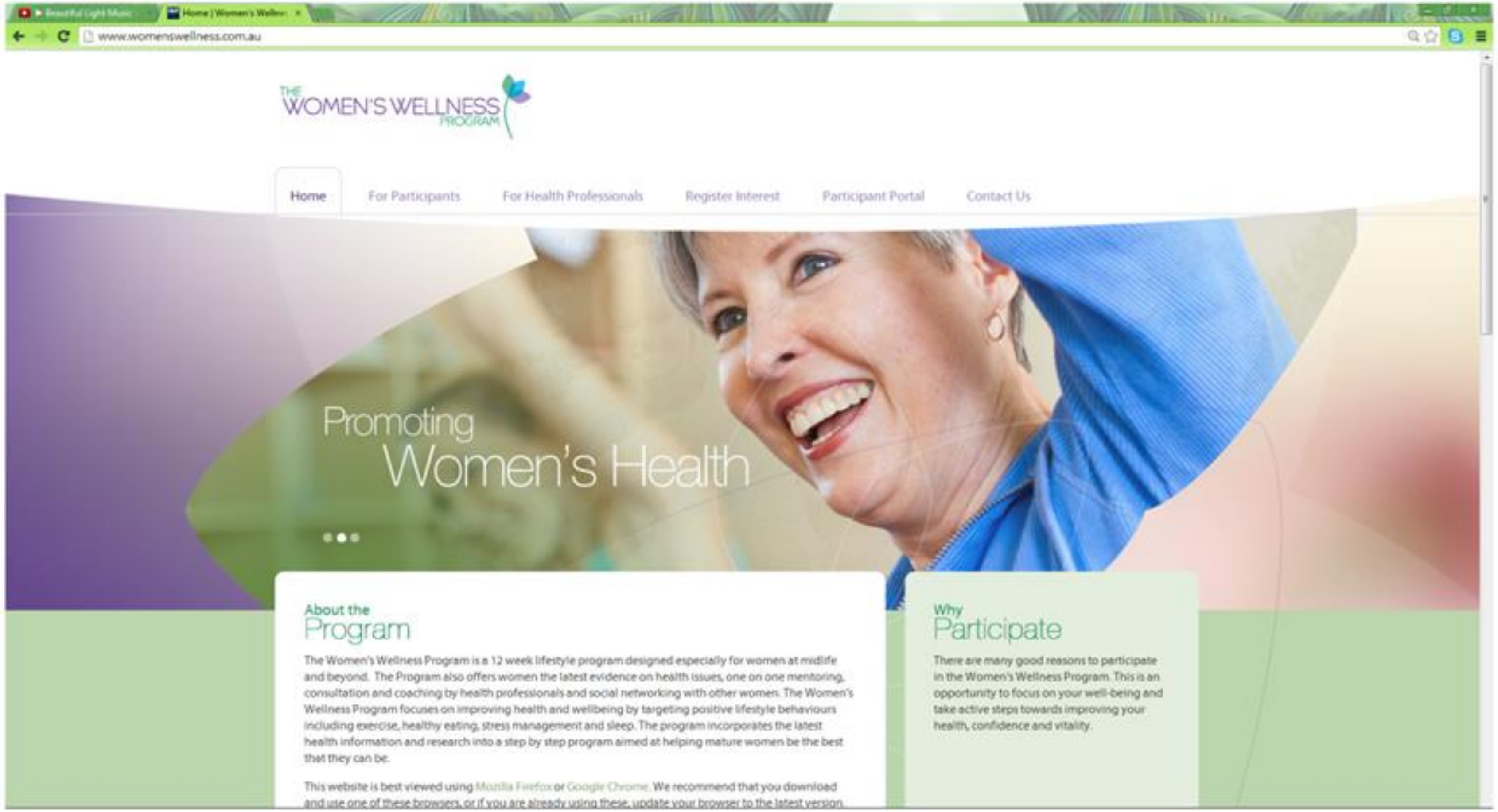

www.womenswellness.com.au

Program E-book

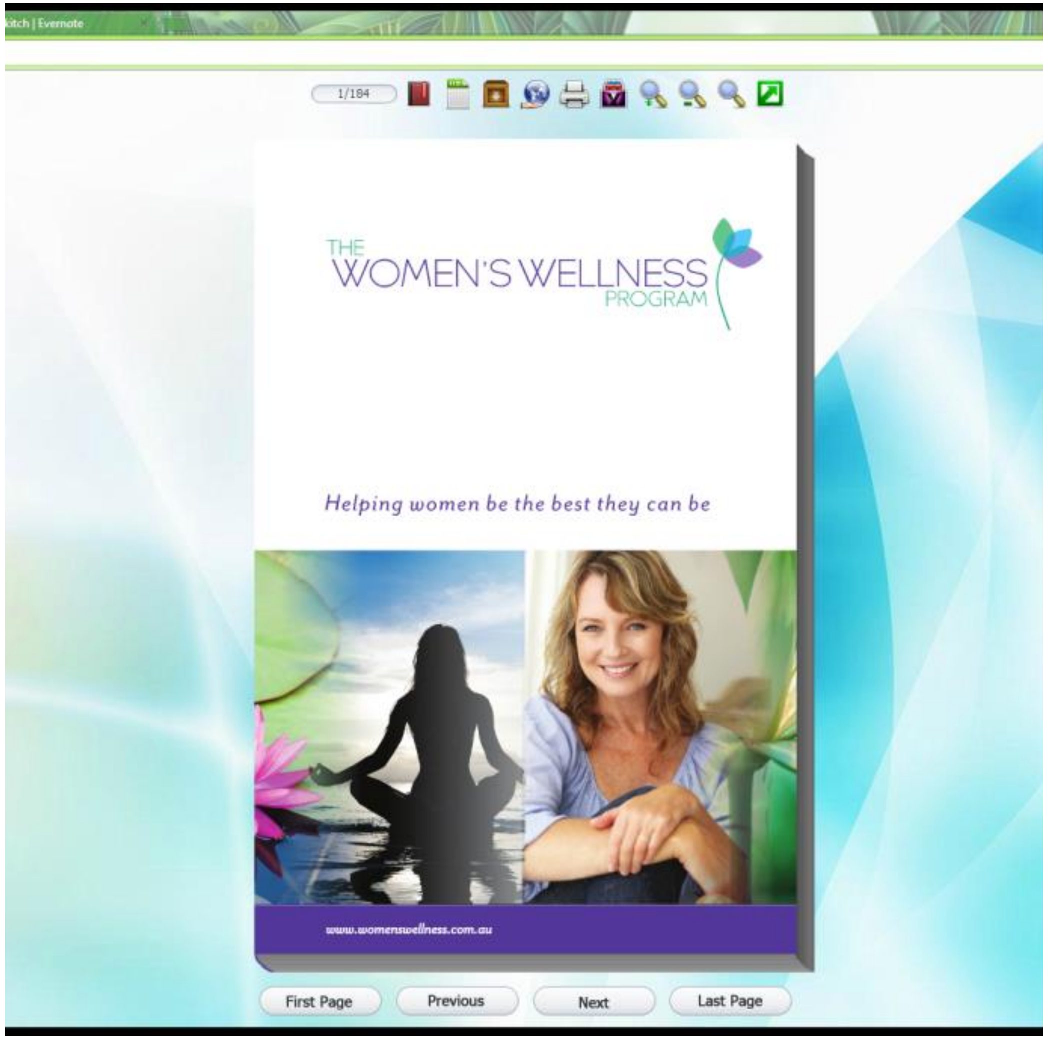

Health coaching delivered by Registered Nurses

- Registered nurse trained to deliver the intervention
- Personalised discussion and support
- SMART goal setting
- 0 weeks, 4 weeks, 8 weeks and 12 weeks

Intervention delivery modes

Group A	**Online independent**<br>• Hard copy of program book, access to website, administrative and technical support
Group B	**Face to face supported with health professional consultations x 4**<br>• Hard copy of program book, delivery in health clinics x 4
Group C	**Online supported with virtual health professional consultations x 4**<br>• Hard copy of program book, access to website, individual consultations through website

For further information contact:

a.mcguire@griffith.edu.au
